# Text-Book of Medical Jurisprudence and Toxicology

**Published:** 1891-11

**Authors:** 


TEXT-BOOK OF MEDICAL JURISPRUDENCE AND TOXICOLOGY. By JOHN J. REESE, M. D., Professor of Medical Jurisprudence and Toxicology in the University of Pennsylvania; late President of the Medical Jurisprudence Society of Philadelphia; Honorary Member of the New York Academy of Anthropology; Corresponding Member of the New York Medico-legal Society, etc. Third edition, revised and enlarged. Small octavo, pp. xvi.—666. Philadelphia: P. Blakiston, Son & Co. 1891.

After a careful perusal of its pages, we lay this volume aside with the profound impression that it should be in the hands of every medical student, physician and lawyer.

The demand for a third edition within two years is a very flattering testimonial of the high esteem in which this work is held by the profession at large.

While the book on the whole is excellent, and we do not hesitate for a single instant to recommend it to the profession, still there are some things in which we do not agree with the author. For example, we do not approve of the administration of bicarbonate of potassium or sodium in cases of mineral acid poisoning. We consider this method of treatment dangerous and liable to lead to rupture of the stomach walls.

In addition to what the author has to say on the treatment of cases of mercurial poisoning, we would add "continue the administration of albumin (white of egg) for one week after the acute symptoms have disappeared."

Under the heading "Poisoning by Opium—Treatment," the author makes no mention of "forced respiration," a method of treatment which has proven efficient in a number of apparently hopeless cases of opium poisoning.

We make these remarks not for the purpose of depreciating in any way the value of this book, but for the purpose of pointing out what we consider are defects in an otherwise excellent volume.

The task of the reviewer is certainly not an enviable one, and we appreciate most fully the difficulties which he has to meet. But we also feel that a review which has been written after a careful perusal of a book, and in which the good points are mentioned and the bad ones called attention to, is of far greater value to the publisher, author, and public in general than one written from the preface alone. J. A. M.

